# Pan-cancer screening, bioinformatics analysis, and experimental validation identify TEX10 as a key biomarker driving OSCC progression

**DOI:** 10.3389/fgene.2026.1750062

**Published:** 2026-06-08

**Authors:** Ruonan Sun, Jiahong Zhao, Jie Guo, Linyu Jin, Jilun Liu, Hui Xu, Yunfang Bai

**Affiliations:** 1 Department of Stomatology, The Second Hospital of Shijiazhuang, Shijiazhuang, Hebei, China; 2 Department of Stomatology, The Fourth Hospital of Hebei Medical University, Shijiazhuang, Hebei, China; 3 Department of Oral Surgery, The Second Hospital of Hebei Medical University, Shijiazhuang, Hebei, China; 4 Department of Emergency, The Fourth Hospital of Hebei Medical University, Shijiazhuang, Hebei, China

**Keywords:** biomarkers, cells cycle, immune microenvironment, oral squamous cell carcinoma, TEX10

## Abstract

**Background:**

TEX10 is a nuclear protein involved in chromatin remodeling. Its pan-cancer diagnostic, prognostic, and immunotherapeutic value has not been systematically evaluated, and its role in oral squamous cell carcinoma (OSCC) remains unclear.

**Methods:**

We employed TCGA and GTEx data for pan-cancer analysis of TEX10’s prognostic and immune associations, along with OSCC-specific clinical, diagnostic and survival correlations. Protein-protein interaction (PPI) network, GO/KEGG enrichment analysis and *in vitro* assays assessed its biological roles and effects on viability, migration, invasion, apoptosis and cell cycle.

**Results:**

TEX10 was upregulated in 15 cancer types and served as a prognostic biomarker for multiple cancers. It was associated with dysregulation of the tumor immune microenvironment and significantly enriched in pathways including cell cycle and DNA replication. In oral squamous cell carcinoma, high TEX10 expression positively correlated with advanced clinical stage, TNM stage, and smoking history, demonstrating high diagnostic accuracy and prognostic value. *In vitro* experiments confirmed that TEX10 knockdown suppressed cell viability, migration, and invasion, promoted apoptosis. Similarly, TEX10 knockdown induced G0/G1 phase cell cycle arrest by downregulating Cyclin D1, CDK4, and upregulating p21.

**Conclusion:**

TEX10 plays an important role in cell cycle progression in OSCC, showing promise as a diagnostic and prognostic biomarker and therapeutic target.

## 1 Introduction

Globally, oral cancer ranks sixth in frequency and is associated with a relatively high mortality rate compared to other cancers. The World Health Organization (WHO) reports that more than 300,000 new oral cancer cases are diagnosed worldwide annually, accounting for 2%–3% of all malignant tumors (Güneri and Epstein, 2014). In 2020, a total of 377,713 cases of oral squamous cell carcinoma (OSCC) were reported globally. The Global Cancer Observatory forecasts an approximate 40% increase in OSCC incidence by the year 2040 (Tan et al., 2023). Between 1990 and 2017 in China, new OSCC cases surged by 280.0%, with age-standardized incidence rates rising 79.7%, while deaths increased by 196.8%, and age-standardized mortality grew by 29.0% (Yang et al., 2021). Clinically, most OSCC patients remain asymptomatic in early stages; they often seek medical attention only after experiencing pain, bleeding, or palpable oral or cervical masses, including lymph node metastasis signs (Silverman et al., 2010; Abati et al., 2020). Therefore, early diagnosis and high mortality remain core challenges in OSCC management. Developing novel and effective diagnostic and therapeutic strategies is an urgent priority.

TEX10 is a gene related to spermatogenesis and belongs to the chromatin remodeling protein family (Yuan et al., 2025). This protein is involved in key biological functions, especially gene expression regulation and chromatin remodeling. TEX10 has been reported to contribute to the development of hepatocellular carcinoma (HCC), pancreatic cancer, bladder cancer, and esophageal cancer (Xiang et al., 2018; Liu et al., 2021; Luo et al., 2021; Xiang et al., 2019). By promoting cancer stem cell-like traits, activating STAT3, and increasing resistance to chemotherapy, TEX10 supports tumor progression in HCC (Xiang et al., 2018). In pancreatic cancer, the miR-30b-5p/TEX10 axis regulates cell proliferation, invasion, and self-renewal, closely associated with Wnt/β-catenin signaling (Liu et al., 2021). TEX10 influences bladder cancer development by regulating XRCC6, enhancing Wnt/β-catenin signaling, and modulating DNA repair processes (Luo et al., 2021). TEX10 facilitates epithelial-mesenchymal transition in esophageal cancer by modulating the Wnt/β-catenin pathway, impacting tumor stemness and metastatic potential (Xiang et al., 2019). Despite TEX10’s crucial roles in multiple cancers, its role in OSCC remains insufficiently characterized, underscoring the need for further investigation.

This study employed comprehensive bioinformatics approaches to investigate the expression patterns, prognostic significance, and immune infiltration characteristics of TEX10 across multiple cancers. We systematically evaluated the expression profile, early diagnostic potential, prognostic value, and associated signaling pathways of TEX10 in OSCC. Experimental validations were conducted to assess the effects of TEX10 on OSCC cell viability, migration, and invasion, as well as its regulatory roles in apoptosis and cell cycle progression during tumorigenesis. Our findings elucidate the critical functions of TEX10 in OSCC pathogenesis and progression, providing new biomarkers and a theoretical foundation for early diagnosis, prognosis assessment, and therapeutic development in OSCC.

## 2 Materials and methods

### 2.1 TEX10 expression variability and its clinical prognostic value in pan-cancer

This study analyzed the differential expression of TEX10 across various cancer types using the BioWinford Platform and TIMER2.0 (http://timer.comp-genomics.org/). To validate the expression patterns, we further utilized GEO datasets across multiple cancer types, including ACC (GSE19776), BLCA (GSE133624), BRCA (GSE59246), CESC (GSE9750), CHOL (GSE89749), COAD (GSE44076), DLBC (GSE147986), ESCA (GSE26886), GBM (GSE109857), HNSC (GSE13399), KICH (GSE15641), KIRC (GSE15641), KIRP (GSE15641), LAML (GSE63270), LGG (GSE35158, GSE285290), LIHC (GSE233422), LUAD (GSE229705), LUSC (GSE67061), MESO (GSE112154), OV (GSE26712), PAAD (GSE211398), PCPG (GSE19422), PRAD (GSE103512), READ (GSE50117), SARC (GSE241095), SKCM (GSE46517), STAD (GSE208099), TGCT (GSE3218), THCA (GSE33630), THYM (GSE79978), UCEC (GSE32507), UCS (GSE32507), UVM (GSE211763), and OSCC (GSE186775, GSE227919). Additionally, we conducted a clinical prognosis analysis of TEX10 using the BioWinford Platform, focusing on overall survival (OS), progression-free survival (PFS), and disease-free interval (PFI). The objective of this analysis was to assess the correlation between TEX10 expression and prognostic outcomes across 33 cancer types. The databases integrated data from TCGA and GTEx, allowing for more complex analyses.

### 2.2 Differential expression analysis of TEX10 in OSCC

The BEST online tool (https://rookieutopia.hiplot.com.cn/app_direct/BEST/) was employed to determine TEX10 expression levels across multiple OSCC datasets and the TCGA database. Furthermore, patients were classified based on other clinical features, such as tumor TNM stages, overall stage, and smoking status, to analyze TEX10 expression in OSCC.

### 2.3 Diagnostic and prognostic analysis of TEX10 in OSCC

Using the Xiantao Academic Tool (https://www.xiantaozi.com/) (Fang et al., 2025; Zhang et al., 2025; Wu et al., 2024), we evaluated the diagnostic and prognostic value of TEX10 in OSCC. For diagnostic assessment, receiver operating characteristic (ROC) curve analysis was performed to determine the ability of TEX10 expression to distinguish OSCC tissues from normal tissues, and the area under the curve (AUC) with 95% confidence intervals (CIs) was calculated. For prognostic evaluation, Kaplan–Meier survival analysis was conducted to compare survival outcomes between high- and low-TEX10 expression groups, including OS, DSS, and PFI. Statistical significance was assessed using the log-rank test, and hazard ratios (HRs) with 95% CIs were calculated. All analyses were performed using the default parameters implemented in the Xiantao Academic platform.

### 2.4 Functional enrichment analysis of TEX10 in OSCC

GeneMANIA (https://genemania.org/) is an online tool for gene function prediction and analysis that explores gene interactions. We used this database to identified genes related to TEX10 and create a protein-protein interaction (PPI) network to reveal patterns of association among gene products. Additionally, using the BEST online tool, we conducted Gene Ontology (GO) analysis to examine biological processes, cellular components, and molecular functions. Subsequently, leveraging the KEGG database, pathway enrichment analysis was conducted to explore signaling and metabolic pathways connected to TEX10.

### 2.5 Cell culture

The HOK cell line, originally frozen at passage 3, was sourced from the Shanghai Cell Bank at the Chinese Academy of Sciences. Cells were cultured in complete DMEM/F12 medium supplemented with 10% heat-inactivated fetal bovine serum (FBS; Gibco) and 1% Penicillin-Streptomycin. To facilitate cell attachment, plates were pre-coated with type I collagen prior to seeding. Cultures were maintained in a humidified incubator at 37 °C with 5% CO_2_. The CAL27, SCC9, and SCC25 cell lines, all obtained at passage five from the Shanghai Cell Bank, were cultured under standardized conditions. Specifically, CAL27 and SCC9 cells were maintained in DMEM medium supplemented with 10% fetal bovine serum (FBS) and 1% Penicillin-Streptomycin. In contrast, SCC25 cells were cultured using RPMI-1640 medium (Gibco) containing the same supplements. Cell morphology and adherence were observed daily under a microscope, and only cells with passage numbers not exceeding P10 were used to ensure consistency. Subculturing was performed once cultures reached 70%–80% confluence, employing 0.25% trypsin-EDTA for detachment to preserve cell viability and reproducibility.

### 2.6 Cell transfection

To interfere with TEX10 expression, we designed several siRNA candidates based on the TEX10 mRNA sequence (NM_001142449.2) using the online siRNA design tool, Invitrogen BLOCK-iT™ RNAi Designer. Specificity was ensured through BLAST analysis to avoid off-target effects. Ultimately, two siRNA sequences with high interference efficiency (si-TEX10#1 and si-TEX10#2) were selected. Cells, cultured in logarithmic growth phase within 6-well plates, were transfected with the chosen siRNAs according to the experimental groups using Lipofectamine™ 3000 reagent (Invitrogen) following the manufacturer’s protocols. To determine TEX10 mRNA expression, total RNA was collected 48 h after transfection and assessed by quantitative reverse transcription polymerase chain reaction (qRT-PCR). Total proteins were similarly extracted for the analysis of TEX10 protein expression by Western blotting. The interference efficiency of each siRNA was evaluated against the reference groups (untreated and si-NC), with the most effective sequence selected for subsequent functional experiments.

### 2.7 qRT-PCR

Using TriQuick Reagent (Solab, Beijing), total RNA was extracted from OSCC and normal oral epithelial cells. RNA purification involved chloroform extraction, precipitation with isopropanol, and a final wash with 75% ethanol. The quantity and purity of RNA were measured using a spectrophotometer. Qualified RNA samples were reverse-transcribed to cDNA with the SureScript First-Strand cDNA Synthesis Kit. Wuhan JinKaiRui Bioengineering Co., Ltd. synthesized the primers employed in qRT-PCR. The TEX10 primer sequences are listed below: TEX10 forward, 5′-TGG​CCG​TAG​CAG​CAT​ATT​GC-3′; TEX10 reverse, 5′-TGA​GAA​GTG​AGT​CTC​CGA​TTA​GG-3′; GAPDH forward, 5′-ACA​ACT​TTG​GTA​TCG​TGG​AAG​G-3′; GAPDH reverse, 5′-GCC​ATC​ACG​CCA​CAG​TTT​C-3′.

### 2.8 Western blot analysis

The original culture medium from each group was discarded, and the cells were washed once with 1× PBS. Subsequently, 1000 μL of RIPA buffer was added to each well, and the cells were incubated with shaking at 4 °C for 30 min. The resulting cell lysate was collected into 1.5 mL centrifuge tubes. Protein concentration was determined using the BCA assay, with a standard curve generated from known standards. Absorbance was measured at 595 nm using a spectrophotometer to calculate protein concentration. Equal amounts of protein (30 μg) were mixed with 5× loading buffer containing β-mercaptoethanol, boiled for 5 min, and immediately placed on ice for 5 min. Samples were then loaded onto a 10% SDS-PAGE gel for electrophoresis. Following separation, proteins were transferred to a PVDF membrane at 4 °C using a constant current of 350 mA for 2 h in transfer buffer. The membrane was blocked with 5% non-fat dry milk at room temperature for 2 h, then incubated overnight at 4 °C with primary antibody. After washing the membrane four times for 5 min each with 1× TBST, it was incubated at room temperature for 1.5 h with an HRP-conjugated secondary antibody. The membrane was placed in Western Lightning™ chemiluminescent substrate for color development for 30 s and immediately scanned for imaging. Band intensity was analyzed quantitatively using ImageJ software. Specific information of primary and secondary antibodies: anti-Tex10 (1: 1000, Thermo Fisher, 720,257), anti-GAPDH (1: 1000, ProteinTech, 10494-1-AP),anti-p21 (1: 1500, Santa Cruz Biotechnology, Cat#sc-397), anti-CDK4 (1: 1500, Santa Cruz Biotechnology, Cat#sc-260), anti-Cyclin D1 (1: 1500, Santa Cruz Biotechnology, Cat#sc-753), anti-MSH2(1: 1000, CST, D24B5), anti-RAD51 (1: 2000, Abcam, ab63801), anti-PCNA(1: 1000, Abcam, EPR3821), goat anti-rabbit IgG (1: 5000, Proteintech, SA00001-2).

### 2.9 CCK-8 cell viability assay

A seeding density of 1 × 10^4^ cells per well was used for plating in 96-well plates, followed by overnight incubation. Logarithmically growing cells were collected initially, with the concentration of the resulting cell suspension then modified. Following this adjustment, 100 μL aliquots of the suspension were placed into each well, ensuring the cellular density per well remained within 1000–10,000 cells. Incubation of cells occurred at 37 °C with 5% CO_2_ for a 24 h period, while the length of this process was adjusted based on the specific cell type and count. After 24 h elapsed, each well received 10 μL of CCK-8 solution. Following a further 1-h incubation period, absorbance at 450 nm was quantified with a microplate reader to determine cell viability.

### 2.10 Wound healing assay

After cells reached near confluence, a scratch was created using a pipette tip. Cells were washed three times with PBS, followed by incubation in serum-free medium to minimize the influence of cell proliferation on migration. Incubation of cells occurred at 37 °C under 5% CO_2_ conditions, and image acquisition was performed at 0 h and 24 h. Scratch width was measured using image analysis software to calculate migration distance and healing rate.

### 2.11 Transwell assay

After digestion, the cells were resuspended in serum-free medium and plated into the upper chamber of a Transwell insert, with the lower chamber containing medium that included serum. The cells were placed in a 37 °C incubator for a 24 h incubation. Post-incubation, cells inside the upper chamber were wiped off with a cotton swab, the chamber was rinsed with PBS, and the cells were fixed in 4% paraformaldehyde for 30 min. A 15-min crystal violet staining step was then conducted. Finally, the membrane was affixed to a slide and observed under a microscope (20×) to assess cell migration.

### 2.12 Cell apoptosis and cycle analysis

For apoptosis analysis, cells (5 × 10^4^–10^5^) were collected, resuspended in 195 µL of Annexin V-FITC binding buffer, and stained with 5 µL Annexin V-FITC and 10 µL propidium iodide (PI) for 15 min at room temperature in the dark. For cell cycle analysis, an aliquot of cells (2 × 10^5^–10^6^) was fixed in pre-cooled 75% ethanol overnight at 4 °C, then washed and stained with 1 mL of DNA staining solution containing PI for 30 min in the dark. Both assays were analyzed using a BD FACSVerse flow cytometer (BD Biosciences, Canada), with apoptosis measured immediately after staining and cell cycle distribution assessed at low instrument acquisition speed.

### 2.13 Statistical analysis

All statistical tests were performed using R (version 4.2.1) and GraphPad Prism software (versions 9.0). Values for each sample were from at least three biologically independent experiments with at least three technical replicates. All data in this study are displayed as the mean ± standard deviation (SD). For comparisons between two groups, a paired t-test was applied to data that met the assumptions of normality and variance homogeneity. Comparisons of three or more groups were analyzed using one-way ANOVA, with Tukey’s HSD *post hoc* test employed for subsequent multiple comparisons. Statistical significance was established at a p-value of less than 0.05. *P < 0.05, **P < 0.01, ***P < 0.001.

## 3 Results

### 3.1 Differential expression of TEX10 in pan-cancer

**FIGURE 1:**
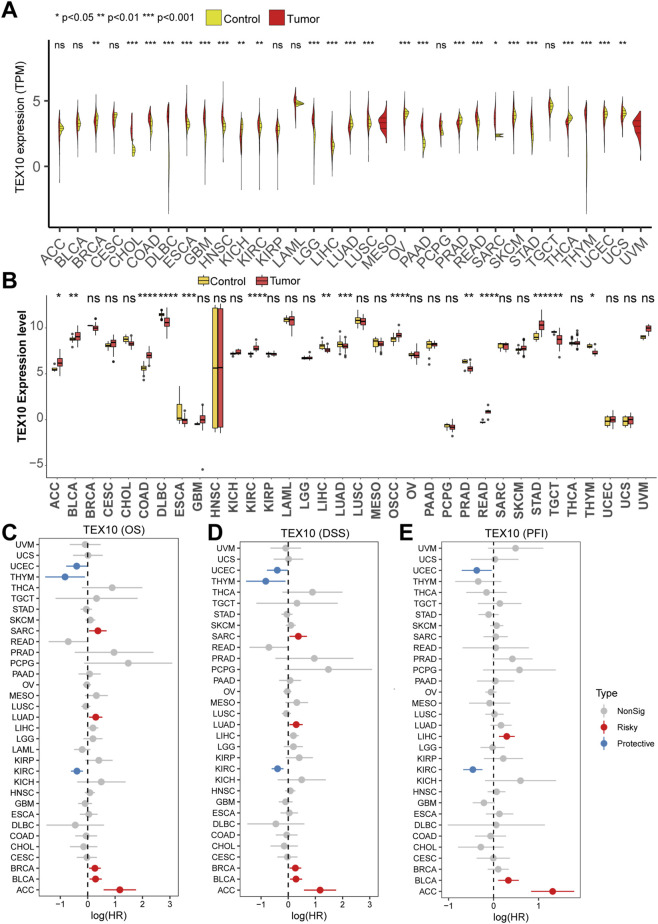
Differential expression analysis and prognostic analysis of TEX10 in pan-cancer. **(A)** TEX10 mRNA expression levels between normal and tumor tissues based on TCGA and GTEx databases. **(B)** Validation of TEX10 expression patterns using independent GEO datasets across multiple cancer types. **(C–E)** The forest plots show the results of univariate COX regression analysis of TEX10 expression in pan-cancer, including **(C)** OS, **(D)** DSS, and **(E)** PFI. OS, overall survival. DSS, disease-specific survival. PFI, progression-free interval.

Gene expression data from the TCGA and GTEx databases were analyzed using the TIMER platform to compare TEX10 mRNA levels between tumor and normal tissues. TEX10 expression was significantly elevated in 15 cancer types, including cholangiocarcinoma (CHOL), colon adenocarcinoma (COAD), diffuse large B-cell lymphoma (DLBC), esophageal carcinoma (ESCA), glioblastoma multiforme (GBM), head and neck squamous cell carcinoma (HNSC), kidney renal clear cell carcinoma (KIRC), brain lower grade glioma (LGG), liver hepatocellular carcinoma (LIHC), lung squamous cell carcinoma (LUSC), pancreatic adenocarcinoma (PAAD), rectum adenocarcinoma (READ), sarcoma (SARC), stomach adenocarcinoma (STAD), and thymoma (THYM) (P < 0.05). Conversely, it was markedly downregulated in nine cancers—breast invasive carcinoma (BRCA), kidney chromophobe (KICH), lung adenocarcinoma (LUAD), ovarian serous cystadenocarcinoma (OV), prostate adenocarcinoma (PRAD), skin cutaneous melanoma (SKCM), thyroid carcinoma (THCA), uterine corpus endometrial carcinoma (UCEC), and uterine carcinosarcoma (UCS) (P < 0.05) (Figure 1A).

To further validate these expression patterns using independent public datasets, we examined TEX10 expression across multiple cancer types using GEO datasets. The results revealed that TEX10 was significantly upregulated in ACC, BLCA, COAD, KIRC, OSCC, PAAD, and STAD, whereas it was downregulated in DLBC, ESCA, LIHC, LUAD, PRAD, TGCT, and THYM (Figure 1B). Notably, while some cancer types (such as COAD, KIRC, PAAD, STAD) showed consistent expression trends between the TCGA/GTEx and GEO datasets, others exhibited heterogeneity, reflecting potential differences in sample composition, platform characteristics, or tumor context-dependency.

### 3.2 TEX10 is a potential prognostic biomarker for multiple cancers

To determine the clinical relevance of TEX10, its association with patient outcomes was evaluated across multiple cancers. Univariate Cox regression analyses were performed to assess correlations between TEX10 expression and OS, DSS, and PFI. High TEX10 expression was significantly linked to shorter OS in SARC, LUAD, BRCA, BLCA, and ACC, whereas low expression predicted poorer OS in UCEC, THYM, and KIRC (P < 0.05) (Figure 1C). In the case of DSS, higher TEX10 expression was linked to worse outcomes in SARC, LUAD, BRCA, BLCA, and ACC, whereas lower TEX10 levels were associated with poorer DSS in UCEC, THYM, and KIRC (P < 0.05) (Figure 1D). In the PFI analysis, higher TEX10 levels were linked to shorter PFI in LIHC, BLCA, and ACC, whereas lower expression was related to unfavorable PFI in UCEC and KIRC (P < 0.05) (Figure 1E).

### 3.3 Immune infiltration analysis of TEX10 in pan-cancer

**FIGURE 2:**
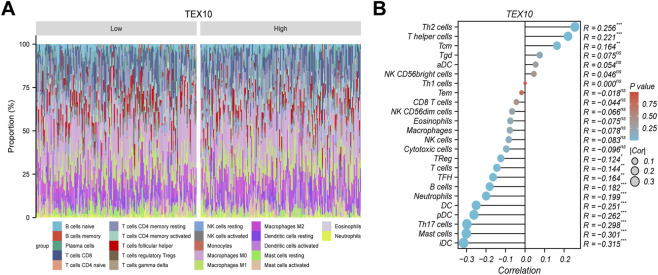
Correlation analysis of pan-cancer immune infiltration. **(A)** Differences in infiltrating immune cell subsets between high TEX10 and low TEX10 groups. **(B)** Correlation between the proportions of TEX10 and 22 infiltrating immune cell subsets in pan-cancer.

The relationship between TEX10 expression and immune cell infiltration was investigated to explore its potential role in the tumor microenvironment. Correlation analysis across multiple cancers revealed that TEX10 expression was significantly associated with the infiltration levels of 22 immune cell types (Figure 2A). Among these, the strongest associations were observed with immature dendritic cells (iDCs), followed by mast cells, Th17 cells, plasmacytoid dendritic cells (pDCs), Th2 cells, conventional dendritic cells, T helper cells, neutrophils, B cells, follicular helper T (TFH) cells, central memory T (Tcm) cells, total T cells, and regulatory T (Treg) cells (Figure 2B).

### 3.4 Upregulation and clinical relevance of TEX10 in OSCC

**FIGURE 3:**
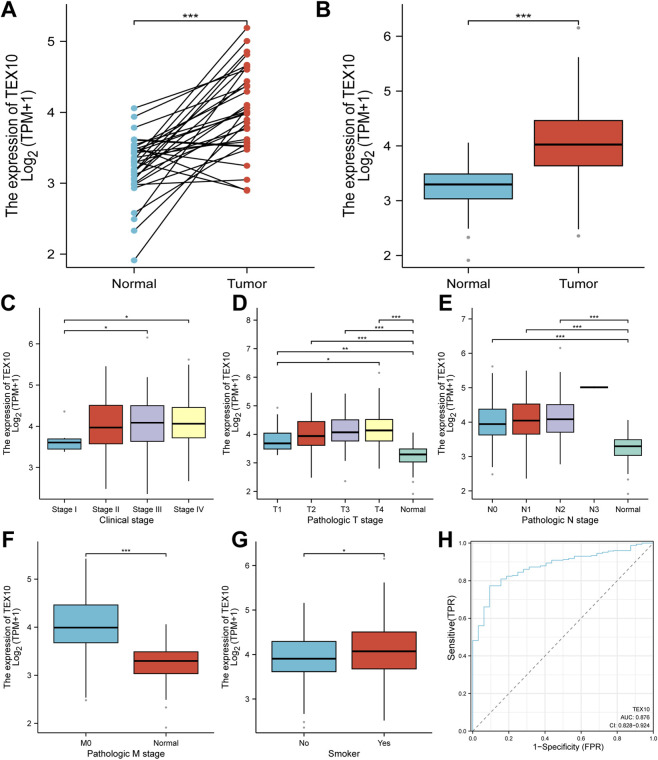
Differential expression, clinical relevance, and diagnostic value of TEX10 in OSCC. **(A,B)** Comparison of TEX10 expression levels between normal and OSCC tissues. **(C)** TEX10 expression across different clinical stages. **(D–F)** Correlation of TEX10 expression with **(D)** T stage, **(E)** N stage, and **(F)** M0 status. **(G)** TEX10 expression stratified by smoking history. **(H)** ROC curve of TEX10 in OSCC.

We next investigated the expression profile and clinical significance of TEX10 in OSCC. Analysis of tumor and normal tissue samples revealed a significant upregulation of TEX10 in OSCC (Figures 3A,B). Further assessment of its association with clinicopathological characteristics showed that TEX10 expression increased progressively with advancing clinical stage (Figure 3C). Elevated TEX10 levels were also positively correlated with larger tumor size and more severe lymph node involvement (Figures 3D,E). The observation that TEX10 remained highly expressed in primary tumors without distant metastasis (M0) compared to normal tissues, which is consistent with the predominance of primary lesions in cohort, and further supports its role in primary tumor progression (Figure 3F). Moreover, TEX10 expression was significantly higher in smokers than in non-smokers (Figure 3G). ROC curve analysis demonstrated that TEX10 had strong diagnostic potential for OSCC, with an AUC of 0.876 (95% CI: 0.828–0.924) (Figure 3H), indicating its reliability as a diagnostic biomarker.

### 3.5 Prognostic value of TEX10 in OSCC

**FIGURE 4:**
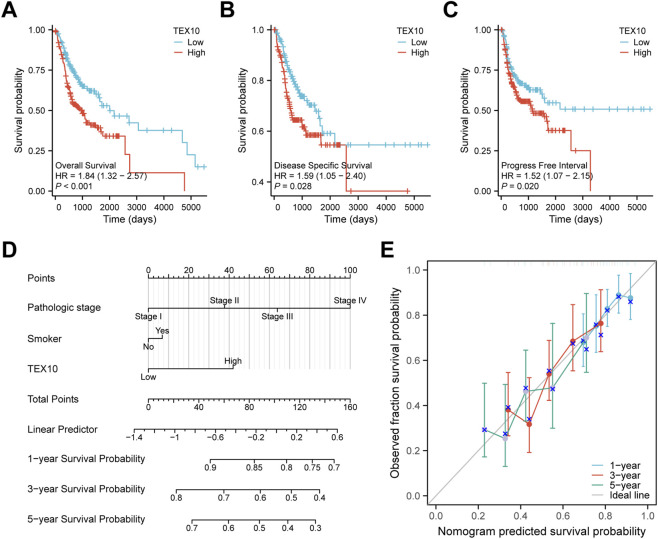
TEX10 as a prognostic biomarker and construction of a clinical nomogram in OSCC. **(A–C)** Patients with high TEX10 expression exhibit significantly worse **(A)** OS, **(B)** DSS, and **(C)** PFI. **(D)** Nomogram for predicting 1-, 3-, and 5-year survival based on pathological stage, smoking status, and TEX10 expression. **(E)** Calibration plots of the nomogram, showing close alignment of predicted survival with actual observed outcomes at 1, 3, and 5 years. OS, overall survival. DSS, disease-specific survival. PFI, progression-free interval.

The prognostic relevance of TEX10 in OSCC was evaluated using Kaplan–Meier survival analysis. High TEX10 expression was significantly associated with poorer outcomes across multiple survival metrics, including OS (HR = 1.84, 95% CI: 1.32–2.57, P < 0.001), DSS (HR = 1.59, 95% CI: 1.05–2.40, P = 0.028), and PFI (HR = 1.52, 95% CI: 1.07–2.15, P = 0.020) (Figures 4A–C). To integrate clinical and molecular variables, a nomogram was constructed incorporating pathological stage, smoking status, and TEX10 expression to predict 1-, 3-, and 5-year survival probabilities (Figure 4D). The model indicated that advanced stage, smoking, and high TEX10 expression were all linked to poorer prognosis. Calibration curves demonstrated strong agreement between predicted and observed survival outcomes, confirming the nomogram’s robust predictive performance over time (Figure 4E).

### 3.6 TEX10 co-expression gene enrichment analysis and PPI network

**FIGURE 5:**
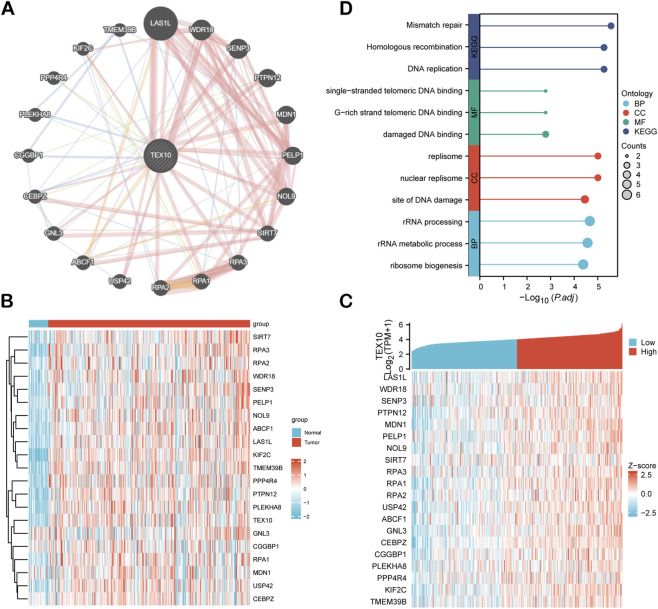
Gene functional set enrichment analysis of TEX10 in OSCC. **(A)** PPI network of TEX10. **(B)** Heatmap of the differential expression of TEX10-associated genes between normal and OSCC samples. **(C)** Correlation analysis between TEX10 expression and its interacting genes in OSCC cohorts. **(D)** Bubble plot showing the significant GO terms and KEGG pathways enriched for TEX10 and its co-expressed genes.

To explore the potential molecular mechanisms of TEX10 in OSCC, a PPI network was constructed using the STRING database. Twenty interacting proteins were identified, including LAS1L, WDR18, SENP3, PTPN12, MDN1, and PELP1, among others (Figure 5A). Expression analyses revealed significant differences in TEX10 and its co-expressed genes between normal and OSCC tissues (Figure 5B), as well as between OSCC samples with low and high TEX10 expression (Figure 5C). KEGG pathway enrichment analysis indicated that TEX10-associated genes were primarily enriched in DNA replication, mismatch repair, and homologous recombination pathways. GO enrichment analysis further showed that these genes participated mainly in rRNA processing and ribosome biogenesis (biological processes), were localized to the replisome (cellular component), and were involved in telomere DNA binding and damaged DNA binding (molecular functions) (Figure 5D).

### 3.7 Knockdown of TEX10 suppressed proliferation, migration, and invasion of OSCC cells

**FIGURE 6:**
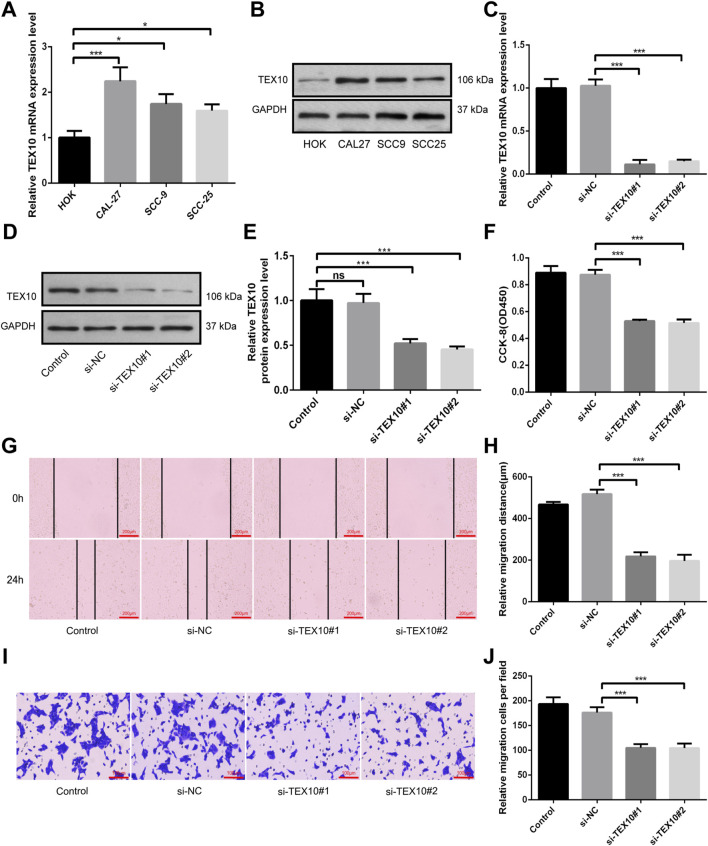
Validation of TEX10 knockdown and its impact on proliferation, migration, and invasion in OSCC cells. **(A,B)** Endogenous TEX10 expression at the **(A)** mRNA and **(B)** protein levels in normal oral epithelial (HOK) and OSCC cell lines. **(C–E)** Knockdown efficiency of TEX10 in CAL27 cells was validated by **(C)** RT-qPCR and **(D,E)** Western blotting. **(F)** Cell viability was assessed by CCK-8 assay after TEX10 knockdown. **(G,H)** Cell migration ability was evaluated by wound healing assay (scale bar: 200 μm). **(I,J)** Cell invasion capacity was determined by Transwell assay (scale bar: 100 μm). Data are presented as mean ± SD from three independent experiments. (n = 3 biological replicates.) *P < 0.05, ***P < 0.001.

To validate the functional role of TEX10 in OSCC, we compared its expression levels between normal oral epithelial cells (HOK) and OSCC cell lines (CAL27, SCC-9, and SCC-25). Both mRNA and protein levels of TEX10 were significantly elevated in OSCC cells (P < 0.05), with the highest expression observed in CAL27 cells (P < 0.001) (Figures 6A,B). Therefore, CAL27 cells were selected for subsequent experiments. TEX10 expression was silenced in CAL27 cells using siRNA, and efficient knockdown was confirmed by RT–qPCR and Western blot analyses (P < 0.001) (Figures 6C–E). Functional assays demonstrated that silencing TEX10 markedly reduced cell viability, as shown by CCK-8 results (P < 0.001) (Figure 6F). Similarly, wound-healing assays revealed significantly impaired migratory capacity in the si-TEX10 group compared with controls (P < 0.001) (Figures 6G,H). Transwell invasion assays further confirmed that TEX10 knockdown substantially decreased the number of invasive cells (P < 0.001) (Figures 6I,J). Collectively, these findings indicate that TEX10 promotes OSCC cell proliferation, migration, and invasion. To further strengthen these findings and exclude potential cell line–specific effects, we additionally performed validation experiments in SCC-9 cells, which exhibited the second-highest TEX10 expression. Efficient knockdown of TEX10 in SCC-9 cells was confirmed by Western blot analyses (Supplementary Figures S1A,B). Consistent with the results observed in CAL27 cells, TEX10 silencing in SCC-9 cells significantly impaired cell migration and invasion, as demonstrated by wound-healing (Supplementary Figures S1C,D) and Transwell invasion assays (Supplementary Figures S1E,F).

### 3.8 Knockdown of TEX10 increased apoptosis and caused G0/G1 phase arrest in OSCC cells

**FIGURE 7:**
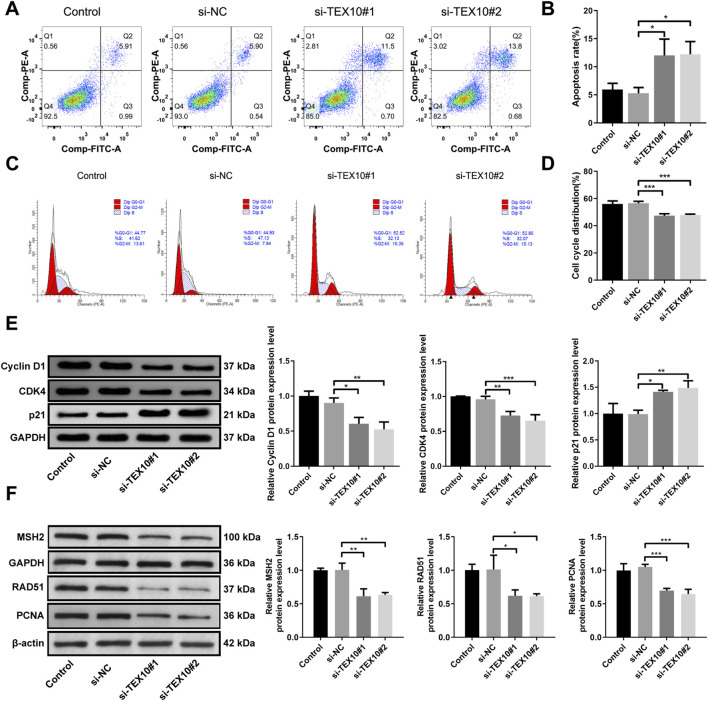
Knockdown of TEX10 induces apoptosis, G0/G1 phase arrest, and suppresses DNA damage repair-related protein expression in CAL27 cells. **(A,B)** Flow cytometric analysis of apoptosis by Annexin V/PI staining after TEX10 knockdown. **(C,D)** Cell cycle distribution analyzed by flow cytometry following TEX10 silencing. **(E)** Protein levels of cell cycle-related molecules were determined by Western blot. **(F)** Protein levels of MSH2/RAD51/PCNA were determined by Western blot. Data are presented as mean ± SD from three independent experiments. (n = 3 biological replicates.) *P < 0.05, **P < 0.01, ***P < 0.001.

To explore whether TEX10 affects cell apoptosis and cycle progression, flow cytometry and Western blot analyses were performed following siRNA-mediated knockdown. TEX10 silencing significantly increased apoptosis compared with the control and si-NC groups (P < 0.05) (Figures 7A,B). Cell cycle analysis revealed an increased proportion of cells in the G0/G1 phase, suggesting that TEX10 depletion induces G0/G1 arrest (Figures 7C,D). Western blot results showed that TEX10 knockdown downregulated the expression of key cell cycle–promoting proteins Cyclin D1 and CDK4 (P < 0.05), while upregulating the cell cycle inhibitor p21 (P < 0.05) (Figures 7E,F). These data indicate that suppression of TEX10 not only promotes apoptosis but also disrupts cell cycle progression by inducing G0/G1 phase arrest in OSCC cells. Similarly, in SCC-9 cells, TEX10 knockdown also increased apoptosis and induced G0/G1 phase arrest, accompanied by decreased Cyclin D1 and CDK4 expression (Supplementary Figures S2A–E).

### 3.9 TEX10 knockdown inhibits the expression of DNA damage repair–related proteins

KEGG enrichment analysis suggested that TEX10 may be involved in DNA mismatch repair, homologous recombination, and DNA replication pathways. To validate this prediction, the expression levels of key proteins in these pathways—MSH2, RAD51, and PCNA—were examined. Western blot analysis showed that the expression of all three proteins was significantly reduced in CAL27 cells following TEX10 knockdown (P < 0.05) (Figure 7F). These findings indicate that TEX10 may influence the progression of oral squamous cell carcinoma by regulating DNA damage repair pathways. Consistent results were observed in SCC-9 cells, where TEX10 silencing also reduced the expression of MSH2, RAD51, and PCNA (Supplementary Figure S2F).

## 4 Discussion

Pan-cancer studies are a frontier in oncology and are crucial for deepening OSCC research. Our results confirmed the overexpression of TEX10 in multiple cancer types, including CHOL, COAD, ESCA, GBM, LGG, LIHC, PAAD, SARC, and THYM. Recent studies also support the link between TEX10 and these cancers (Liu et al., 2021; Xiang et al., 2019; Xiang et al., 2023; Wang et al., 2020; Kuk et al., 2024). We further found that high TEX10 expression correlates with poor prognosis. These findings suggest TEX10 as a potential cancer biomarker. Based on this, we propose TEX10 as a biomarker for OSCC prognosis. OSCC is a common malignancy (Kingsley et al., 2008; Kao and Lim, 2015), and research on therapeutic targets and prognosis is becoming increasingly critical in clinical treatment (Garlet, 2013). Analysis of clinical correlations revealed that TEX10 expression positively correlates with the TNM stage of OSCC. This suggests that TEX10 is activated early in tumor development and increases with disease progression, contributing to growth, invasion, and metastasis. We also observed a strong association between TEX10 expression and history of smoking. Cigarette smoking constitutes a major risk factor for OSCC, and its carcinogenic effect is mainly linked to remodeling of the inflammatory microenvironment (Qian et al., 2022). The upregulation of TEX10 in smokers suggests it may be a key molecular link in tobacco-induced carcinogenesis, accelerating tumor progression. Notably, recent studies reported a close relationship between TEX10 and OSCC, supporting its potential value as a clinical diagnostic marker (Kuk et al., 2024). Building on this, our study further explored the association of TEX10 expression with tumor TNM staging and smoking status to better understand its role and clinical significance in OSCC pathogenesis. Although OSCC treatment has improved (Montero and Patel, 2015), early diagnosis remains challenging, limiting cure rates (van der Waal et al., 2011). The ROC curve results show that the AUC value of TEX10 for OSCC is 0.876, indicating high reliability. Compared to the traditional marker SCC-Ag (AUC = 0.713) (Chen et al., 2013), TEX10 demonstrates superior diagnostic performance and reliability. This highlights its potential for early OSCC diagnosis.

TEX10 is emerging as an important regulatory factor in tumor progression and treatment outcomes (Fanis et al., 2012; Ding et al., 2015; Gentilella et al., 2015; Penzo et al., 2015). In this study, PPI analysis uncovered that TEX10 interacts with immune-related genes such as SENP3 and PELPI. This suggests that TEX10 may influence tumor cells by regulating the expression of these genes, thereby promoting tumor progression. Results from enrichment analysis showed that TEX10 is primarily engaged in essential biological processes such as DNA repair, DNA replication, assembly of nuclear replication complexes, rRNA processing, and ribosome biogenesis. These processes are highly linked with precise cell cycle control. DNA replication is the core event of the S phase (Bournaka et al., 2024), while DNA repair is crucial for the G1/S checkpoint to monitor damage and ensure cycle fidelity (Murray-Nerger et al., 2021). In addition, rRNA processing and ribosome biogenesis provide the biosynthetic capacity required for sustained protein synthesis and rapid tumor cell growth (Baßler and Hurt, 2019). Therefore, dysregulation of these processes may directly influence cell cycle progression and tumor cell proliferation.

Consistent with these bioinformatic predictions, our functional experiments demonstrated that TEX10 knockdown significantly suppressed proliferation, migration, and invasion of OSCC cells while simultaneously increasing apoptosis and inducing G0/G1 phase arrest. Mechanistically, TEX10 knockdown markedly decreased the expression of the cell cycle–promoting proteins CDK4 and Cyclin D1, accompanied by a significant increase in the CDK inhibitor p21. The CDK4/Cyclin D1 complex plays a central role in phosphorylating retinoblastoma (Rb) protein and releasing E2F transcription factors, thereby driving the transition from the G1 to the S phase (Elmetwali et al., 2016). Reduced CDK4 and Cyclin D1 expression after TEX10 knockdown likely disrupts this regulatory axis, preventing efficient G1/S transition. Meanwhile, elevated p21 can directly bind to Cyclin–CDK complexes and inhibit their kinase activity, further blocking cell cycle progression and contributing to G0/G1 arrest (Niculescu et al., 1998). These findings indicate that TEX10 promotes OSCC cell proliferation partly through regulation of the CDK4/Cyclin D1/p21 signaling axis.

Beyond its role in regulating specific oncogenic signaling pathways, accumulating evidence suggests that TEX10 also participates in nucleolar regulatory complexes involved in ribosome biogenesis. TEX10 has been identified as a component of the PELP1-TEX10-WDR18 complex, which localizes to the nucleolus and participates in rRNA processing and maturation of the 60S ribosomal subunit (Finkbeiner et al., 2011). The nucleolus is the central hub for ribosome production, and growing evidence indicates that dysregulation of ribosome biogenesis is closely linked to tumorigenesis because rapidly proliferating cancer cells require increased translational capacity to sustain continuous growth (Wang et al., 2024; Hong et al., 2024). In this context, TEX10 may contribute to tumor progression by coordinating nucleolar ribosome assembly with cellular proliferative programs. Enhanced ribosome biogenesis can increase global protein synthesis and support the expression of cell cycle regulators, thereby facilitating DNA replication and cell cycle progression. Conversely, disruption of nucleolar ribosome assembly has been shown to impair cellular proliferation and trigger cell cycle arrest. These observations suggest that TEX10 may promote OSCC progression by maintaining nucleolar biosynthetic activity and translational capacity required for sustained tumor cell proliferation.

The tumor microenvironment consists of tumor cells along with invasive immune and stromal cells. The infiltration of immune cells exerts a key role in tumor progression and immunotherapy efficacy (Sadeghi Rad et al., 2021; Sadeghi Rad et al., 2020). Our study found TEX10 positively correlates with immunosuppressive cells including Th2 and central memory Tcm cells, notably showing a strong association with Th2 cells. This insight is important for understanding OSCC immune escape mechanisms. Th2 cells are key players in OSCC immune evasion. They secrete factors including IL-4 and IL-10, which dampen CD8^+^ cytotoxic T-cell functions and enhance the immunosuppressive microenvironment (Marple et al., 2017). TEX10’s positive correlation with Th2 cells suggests that elevated TEX10 in OSCC may promote local accumulation of Th2 cells, accelerating immune escape and tumor progression. Likewise, Tcm cells tend to exhibit immunosuppressive phenotypes within the OSCC microenvironment. The correlation between TEX10 and Tcm cells points to further immune imbalance driven by TEX10. This finding offers new directions for OSCC immunotherapy. Targeting TEX10 may modulate Th2 and Tcm cell activities, restoring immune balance and improving therapy. However, more experiments are necessary to unravel the detailed regulatory mechanisms that participate in this process.

However, several limitations should be acknowledged. (1) Although we integrated bioinformatics analyses with *in vitro* functional experiments to explore the role of TEX10 in OSCC, the current evidence remains largely associative and has not yet been validated *in vivo* or in independent patient cohorts/clinical samples. (2) Although our Western blot results confirmed that TEX10 knockdown reduces the expression of MSH2, RAD51, and PCNA—key proteins involved in DNA mismatch repair, homologous recombination, and DNA replication—these findings only provide preliminary evidence of TEX10’s involvement in DNA damage repair pathways. The precise molecular mechanisms by which TEX10 regulates these pathways remain to be further elucidated. (3) Since TEX10 knockdown was found to induce apoptosis in OSCC cells, the observed differences in cell migration and invasion following TEX10 silencing may be secondary to increased cell death rather than a direct functional effect of TEX10 on these processes. Further experiments, such as using apoptosis inhibitors or performing single-cell tracking assays, are needed to dissect the direct role of TEX10 in regulating migration and invasion independently of its effects on cell viability. Future studies should focus on validating these findings in vivo models and independent clinical cohorts, as well as further elucidating the molecular mechanisms underlying TEX10-mediated regulation of DNA damage repair. Specifically, rescue experiments, co-immunoprecipitation assays to identify potential interacting proteins, and pathway-specific functional assays will be required to clarify the regulatory mechanism of TEX10 in OSCC. In addition, apoptosis inhibition or cell-cycle synchronization assays may help distinguish whether TEX10-related effects on migration and invasion are direct or indirect consequences of apoptosis.

Our study highlights the critical role of TEX10 in the pathogenesis of OSCC, highlighting its potential as a prognostic biomarker and therapeutic target, while also laying the foundation for a precision oncology approach targeting its multifaceted functions. The effects of TEX10 on immune cell infiltration, cell cycle progression, apoptosis resistance, and metastatic potential support its relevance in tumor biology.

## 5 Conclusion

In summary, our study identifies TEX10 as a significant pan-cancer biomarker, particularly in OSCC, where it serves as a promising target for therapeutic intervention. This research presents the first comprehensive pan-cancer analysis of TEX10 expression in relation to prognosis and immune infiltration. Through integrated bioinformatics and experimental approaches, we have systematically elucidated the clinical significance and potential functions of TEX10 in OSCC pathogenesis. Our findings not only establish TEX10’s crucial role in tumor progression through regulation of cell cycle and apoptosis, but also provide foundational insights for future investigations into its molecular mechanisms and therapeutic applications.

## Data Availability

The original contributions presented in the study are included in the article/Supplementary Material, further inquiries can be directed to the corresponding author.
