# Supplementary material for: Pan-cancer screening, bioinformatics analysis, and experimental validation identify TEX10 as a key biomarker driving OSCC progression
Source: Front Genet. 2026 Jun 8;17:1750062. doi: 10.3389/fgene.2026.1750062 (PMC13283488; doi:10.3389/fgene.2026.1750062)
Supplement: Supplementary file 1 [file DataSheet1.docx]

**Supplementary Figures:**


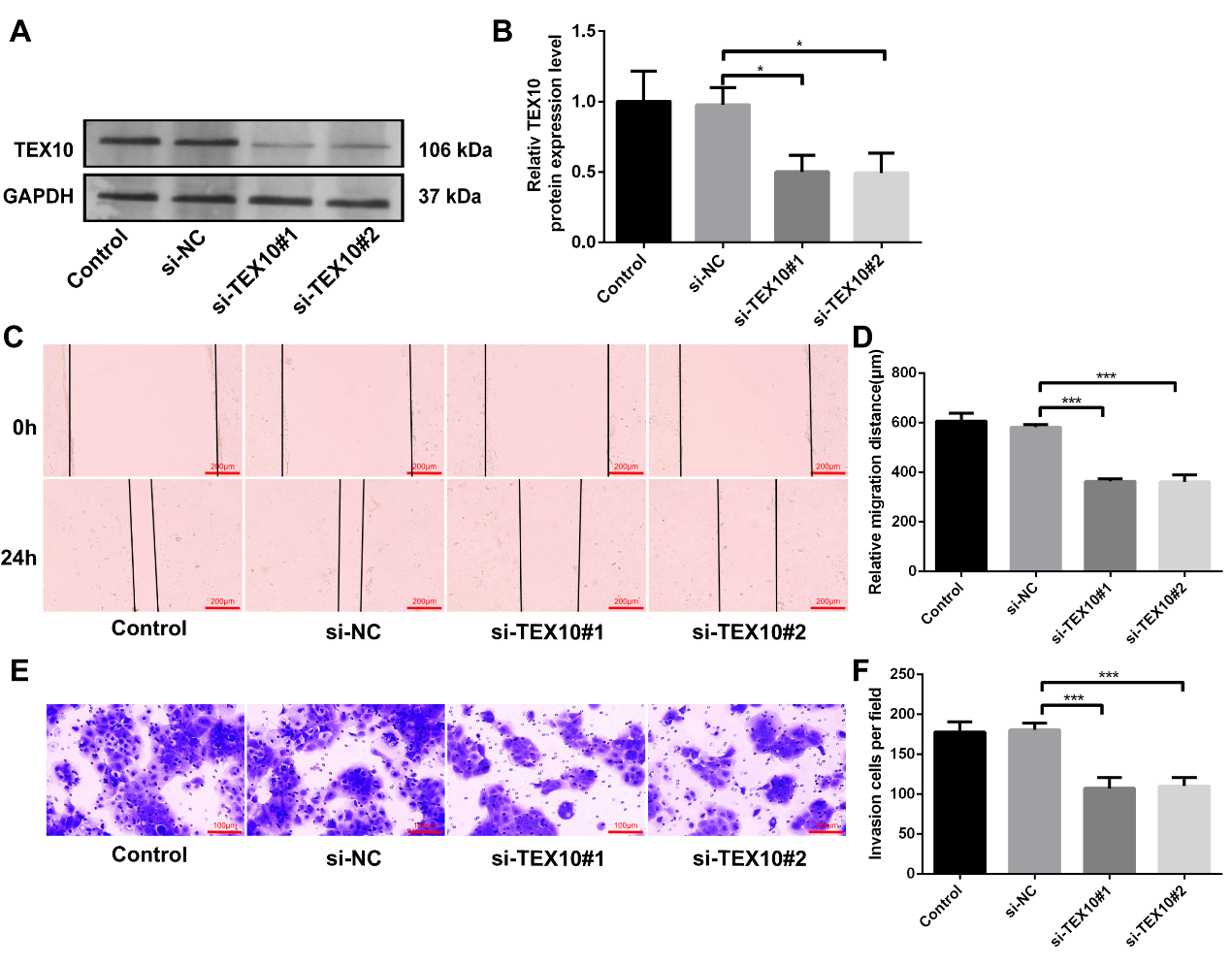


Figure S1. Validation of TEX10 knockdown efficiency and its effects on migration and invasion in SCC9 cells. (A-B) Knockdown efficiency of TEX10 was validated by Western blotting. (C-D) Cell migration ability was evaluated by wound healing assay (scale bar: 200 μm). (E-F) Cell invasion capacity was determined by Transwell assay (scale bar: 100 μm). Data are presented as mean ± SD from three independent experiments. (n = 3 biological replicates.) *P < 0.05, ***P < 0.001.


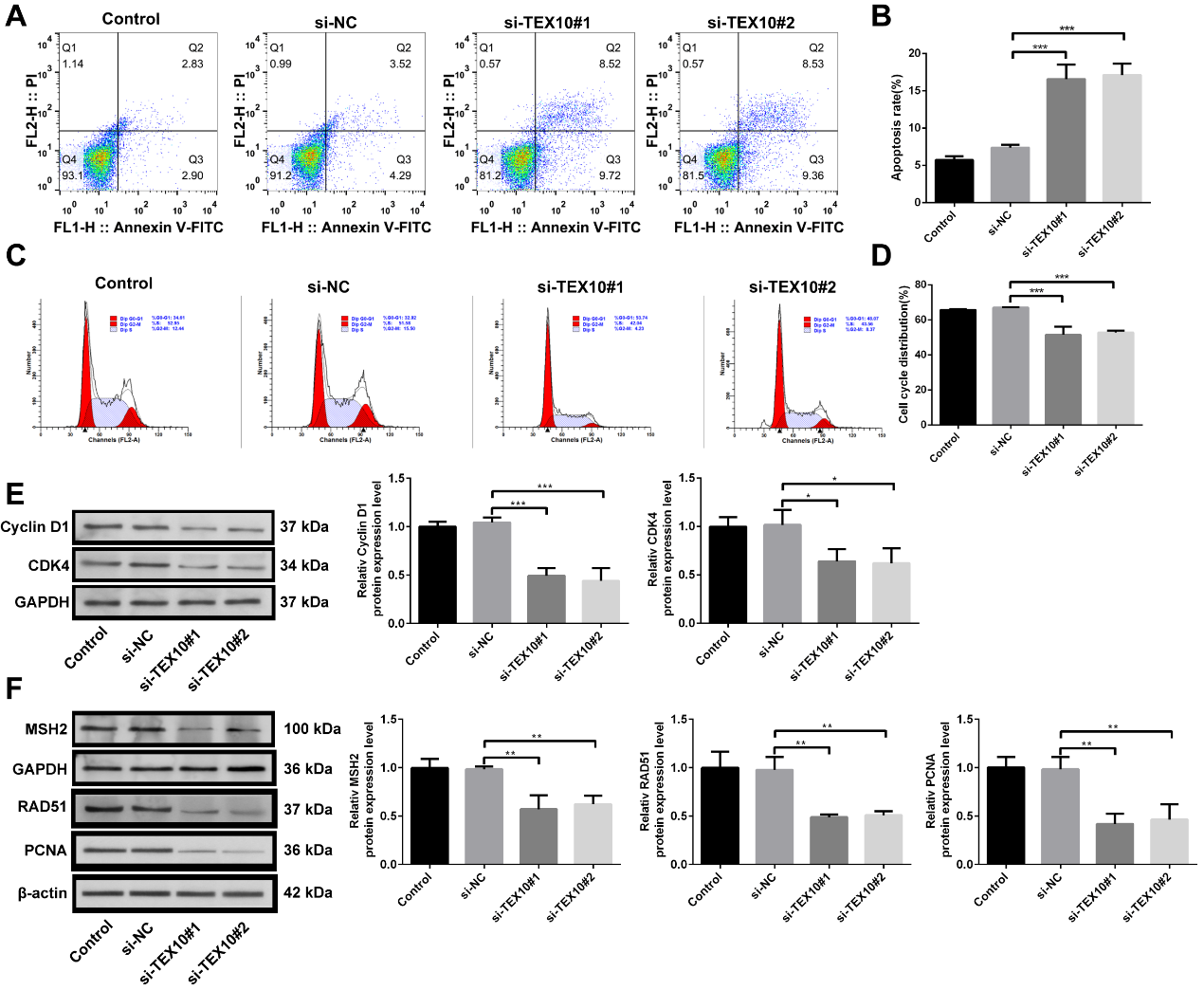


Figure S2. Knockdown of TEX10 induces apoptosis, G0/G1 phase arrest, and suppresses DNA damage repair-related protein expression in SCC9 cells. (A-B) Flow cytometric analysis of apoptosis by Annexin V/PI staining after TEX10 knockdown. (C-D) Cell cycle distribution analyzed by flow cytometry following TEX10 silencing. (E) Protein levels of cell cycle-related molecules were determined by Western blot. (F) Protein levels of MSH2/RAD51/PCNA were determined by Western blot. Data are presented as mean ± SD from three independent experiments. (n = 3 biological replicates.) *P < 0.05, **P < 0.01, ***P < 0.001.
